# Probing the growth and melting pathways of a decagonal quasicrystal in real-time

**DOI:** 10.1038/s41598-017-17821-0

**Published:** 2017-12-12

**Authors:** Insung Han, Xianghui Xiao, Ashwin J. Shahani

**Affiliations:** 1 Department of Materials Science and Engineering, University of Michigan, Ann Arbor, Michigan 48109 United States; 2 X-ray Science Division, Advanced Photon Source, Argonne National Laboratory, Lemont, Illinois 60439 United States

## Abstract

How does a quasicrystal grow? Despite the decades of research that have been dedicated to this area of study, it remains one of the fundamental puzzles in the field of crystal growth. Although there has been no lack of theoretical studies on quasicrystal growth, there have been very few experimental investigations with which to test their various hypotheses. In particular, evidence of the *in situ* and three-dimensional (3D) growth of a quasicrystal from a parent liquid phase is lacking. To fill-in-the-gaps in our understanding of the solidification and melting pathways of quasicrystals, we performed synchrotron-based X-ray imaging experiments on a decagonal phase with composition of Al-15at%Ni-15at%Co. High-flux X-ray tomography enabled us to observe both growth and melting morphologies of the 3D quasicrystal at temperature. We determined that there is no time-reversal symmetry upon growth and melting of the decagonal quasicrystal. While quasicrystal growth is predominantly dominated by the attachment kinetics of atomic clusters in the liquid phase, melting is instead barrier-less and limited by buoyancy-driven convection. These experimental results provide the much-needed benchmark data that can be used to validate simulations of phase transformations involving this unique phase of matter.

## Introduction

Since the discovery of quasicrystals (QCs) by Shechtman in 1984^1,2^, the growth mechanism of QCs has stimulated the curiosity of researchers worldwide due to their unique structure^3–12^. QCs are structures that exhibit long range order and non-crystallographic symmetry elements, while lacking 3D periodicity. Typically QCs have 5-, 8-, 10-, or 12-fold symmetry, which were considered “forbidden” since a motif of these symmetries cannot fill all of 2D plane or 3D space. From the perspective of conventional crystallography, only 2-, 3-, 4-, and 6-fold symmetries can exist in nature. As such, the discovery of QCs has led to a revision in the definition of a crystal by the International Union of Crystallography to a material with discrete diffraction patterns regardless of its periodicity^13^. Worth noting is that QCs exhibit many distinct properties originating from their aperiodic structure: For example, icosahedral QCs have high resistivity, hardness, thermal stability, and low friction, which make them good candidates for surface coatings and catalysts^14–19^.

There are two classes of QCs: 2D and 3D structures. 2D QCs (e.g., Penrose tiling^20^) exhibit an aperiodic order in 2D and a periodic order in the remaining third dimension (“periodic direction”). In the case of decagonal QCs (e.g., 10/mmm), there are two-fold planes (i.e., {10000}) and ten-fold planes (i.e., {00001}). Only the plane with the ten-fold symmetry consists of a quasicrystalline atomic arrangement; perpendicular to this plane is the periodic <00001> direction. On the other hand, the structures of the three-dimensional QCs are quasiperiodic in all three dimensions (e.g., icosahedral QCs). That is, they do not have translational periodicity in any direction. Historically, the discovery of 2D QCs^21^ followed that of 3D QCs^1,2^.

To explain the growth mechanisms of both variants, several models have been proposed, such as ideal tiling models^22,23^ and cluster-based models^24–26^. In ideal tiling models, two or more types of structural units, such as rhombi, are packed without generating gaps or overlaps^22,23^. The tiles follow local matching rules to produce a packed, aperiodic pattern. The Penrose tiling is one of the representative examples. Because the rhombi need to be present in just the right proportion to generate quasiperiodicity, it seems unlikely that the tiling model can explain the growth of real QCs in metallic alloys. Instead, researchers^24–26^ have advanced a cluster-based model, in which QCs are formed by single, repeating clusters or quasi-unit-cells (e.g., the Gummelt decagon^27^). The clusters or quasi-unit-cells may overlap in specific ways to create quasicrystalline order^27^. As several researchers^11,28,29^ have already identified such clusters in QC-forming, supercooled liquids, the picture of a QC growing from the attachment of these clusters at the solid-liquid interface remains a distinct possibility. Unfortunately, experimental validation of these proposed and simulated mechanisms is lacking, especially concerning the growth and melting of metallic QCs. Thus, there are still unanswered questions in terms of the underlying kinetics, surface properties, and defects.

To answer these questions, refs^5–7^ have analyzed the growth of icosahedral QCs *via in situ* X-ray radiography (i.e., projection videomicroscopy). They studied the interfacial velocity of the facets and edges in the icosahedral QC during directional solidification. Based on the results obtained, the researchers suggested that QC growth is in some ways analogous to the kinetics of ledge growth in semiconductors^30–32^. While one can extract some qualitative information from the 2D images, X-ray radiography is prohibitive since most growth models, such as those listed above, make predictions based upon a 3D structure. In a similar sense, Nagao *et al*. have studied grain growth in a decagonal QC specimen using *in situ* high resolution transmission electron microscopy (HRTEM)^33^. This study, too, is limited to the ten-fold {00001} plane, i.e., there is no mention of the interfacial dynamics along the period direction. *In situ* and 3D experimental studies on the growth and melting of QCs from a parent liquid phase have not been demonstrated yet, to the best of our knowledge.

**Figure 1:**
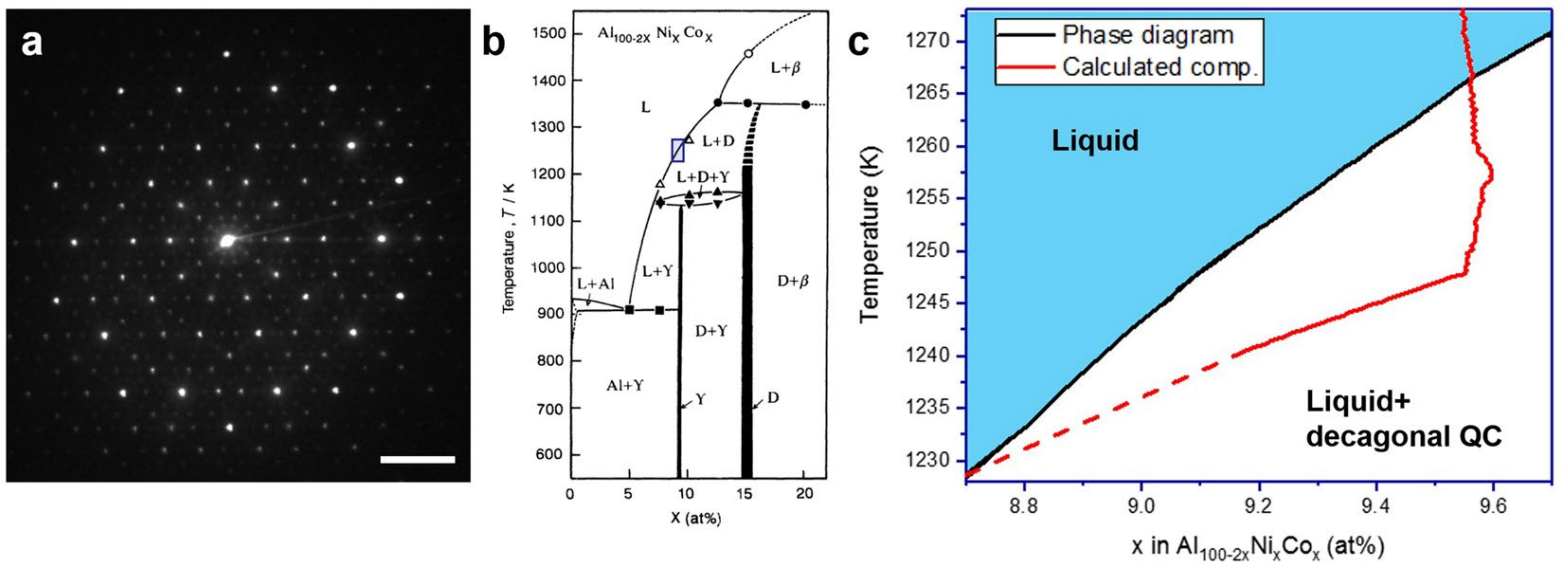
*System-of-interest*. (**a**) Electron diffraction pattern of the solid QC phase, which proves unambiguously the decagonal symmetry of the QC phase in the Al-Ni-Co system. The scale bar measures 2 nm^−1^. (**b**) Partial section of the Al_100−2x_Ni_x_Co_x_ pseudo-binary, equilibrium phase diagram as measured by Yokoyama *et al*.^35^ and reprinted with permission from the Japan Institute of Metals and Materials. $L$ and $D$ indicate the liquid and decagonal QC phases, respectively. (**c**) Calculated, alloy composition within the FOV during the XRT experiment (red) superimposed on the same phase diagram (black). The region plotted in (**c**) corresponds to the blue boxed region in (**b**). The solid QC grows and then melts due to the “pile up” of Al in the liquid phase. The dotted line indicates extrapolated compositions.

Herein, we present our efforts to capture the growth and melting of a decagonal QC *via* four dimensional (i.e., 3D space plus time), synchrotron-based X-ray tomography (XRT). We focus our investigation on the Al-Co-Ni system, which contains a thermodynamically stable decagonal QC phase^34^. As a first step and to prove the existence of decagonal QCs in this system, we recorded the electron diffraction pattern of the stable decagonal QC phase at ambient temperature (Fig. 1(a)). As expected, the diffraction pattern shows sharp reflections and the requisite 10-fold symmetry.

The synchrotron experiment was performed on an alloy of nominal composition Al-9.55at%Ni-9.55at%Co. Our results were obtained by continuously cooling an Al-9.55at%Ni-9.55at%Co rod sample from above its liquidus temperature into the two-phase, decagonal QC plus liquid regime. A partial section of the pseudo-binary Al_100−2x_Ni_x_Co_x_ phase diagram is shown in Fig. 1(b), which is reprinted from Yokoyama *et al*.^35^ Observe that the solidification of the Al-15at%Ni-15at%Co QC is non-congruent, with rejection of Al as growth proceeds. This means that we were able to achieve absorption contrast between the QC and the surrounding liquid phase owing to differences in X-ray absorption^36^.

Although the entire rod sample was cooled during the tomography experiment, only a portion of the sample was imaged (hereafter referred to as the tomographic field-of-view or FOV). This FOV behaves as an open system, exchanging solute (Al, Ni, and Co) with other parts of the rod sample. We observed (Fig. 1(c)) that the alloy composition within the FOV becomes Al-rich as time proceeds. Here, the time-dependent alloy composition was found quantitatively by mapping the variation of intensity in the X-ray projection images to composition (see Methods). The “pile up” of the Al constituent is likely due to a combination of gravity-induced segregation and Al rejection from regions outside the FOV. Eventually, the accumulation of Al in the liquid initiates melting of the QC, by lowering the melting point of the solid-liquid interface. A similar phenomenon of dendrite arm remelting has been reported by several investigators, e.g., ref.^37^. Thus, we are able to measure *both* growth and melting in the same experiment, as the sample enters and exits the two-phase decagonal QC plus liquid regime in Fig. 1(c), respectively. Further experiments are underway to shed light on the effects of transient growth conditions on QC melting.

Following the 4D XRT experiment, we processed the Big Data (1.4 TB in volume) and visualized the morphology of the decagonal QC as a function of time (and hence, temperature) during growth and melting. From these 3D snapshots, we quantified the local interfacial orientation and velocities, and the correlations between the two. These results have made it possible to investigate kinetic phenomena at the QC-liquid interface for the first time, as will be explained in detail below.

## Results

### Three-dimensional reconstructions

**Figure 2:**
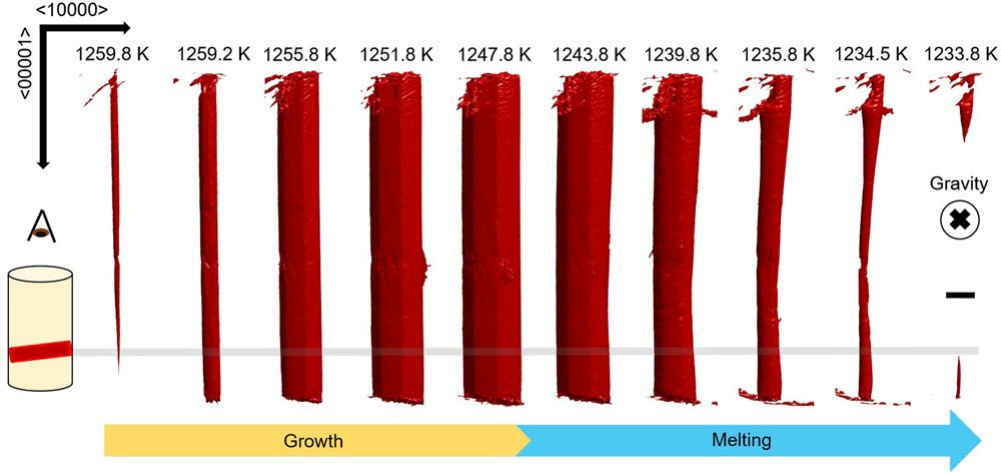
Three dimensional reconstructions (bird’s eye view, see inset) of quasicrystal growth and melting during continuous cooling. The temperature decreases from left to right as a function of reaction time. Temperatures and times are as follows: 1259.8 K (800 sec), 1259.2 K (840 sec), 1255.8 K (1040 sec), 1251.8 K (1280 sec), 1247.8 K (1520 sec), 1243.8 K (1760 sec), 1239.8 K (2000 sec), 1235.8 K (2240 sec), 1234.5 K (2320 sec) and 1233.8 K (2360 sec) respectively. The start of the clock (0 sec) corresponds to the start of the XRT experiment. Scale bar measures 100 µm.

We detected one single QC within the tomographic FOV during continuous cooling. Its evolution at ten representative time intervals is depicted in Fig. 2. The QC grows from the one side to the other side of the Al_2_O_3_ skin that contains the molten alloy (not pictured). The QC is anchored to this oxide skin on both sides and therefore the QC does not sediment to the bottom of the melt. The growth velocity along the periodic direction is approximately over two orders of magnitude greater than the velocity along the aperiodic direction, which corroborates the *ex situ* observations of Gille *et al*.^38^ and Meisterernst *et al*.^39^ The “long axis” parallel to <00001> represents the fast-growing, periodic direction and <10000> represents the set of ten slow-growing, aperiodic directions. When the QC melts, it does not mirror the growth pathway, which is fully faceted and nearly isotropic in the aperiodic <10000> directions. Rather, we observe marked curvature of the solid-liquid interface upon melting. Therefore, the growth and melting processes do not have time-reversal symmetry and hence different physical principles must be invoked in order to explain these different behaviors (see Discussion). Furthermore, we investigated a cross-section of the 3D reconstructed volume. The region selected for analysis is highlighted with a grey line in Fig. 2 and includes a thickness of 20 µm along the periodic direction.

### Analysis of ten-fold plane

**Figure 3:**
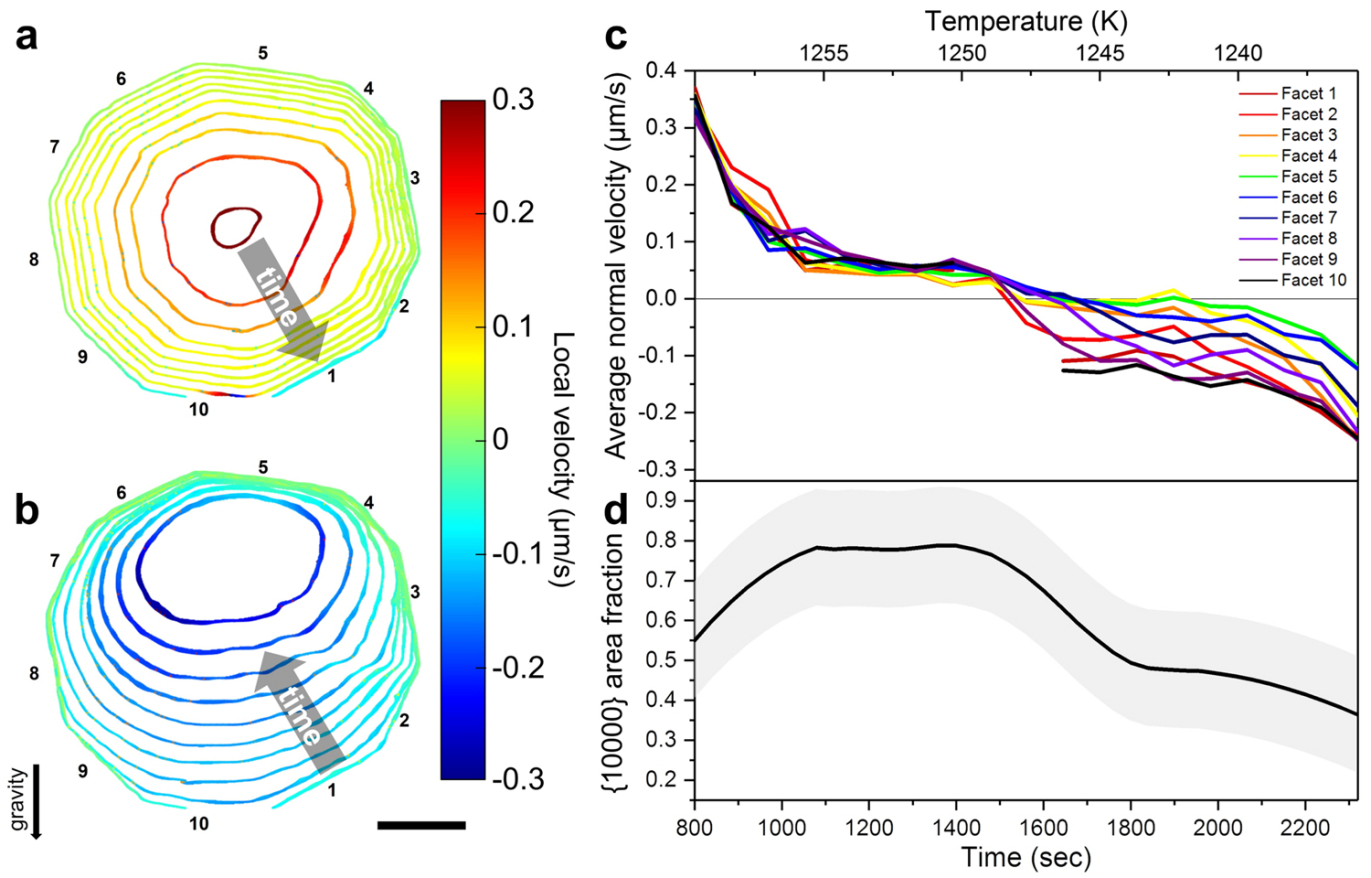
*Evolution of the ten-fold plane* as a function of time for QC (**a**) growth and (**b**) melting. Isochrones of the solid-liquid interface are colored according to their interfacial velocity, which is positive for growth and negative for melting. During growth, the QC develops ten distinct facets (numbered from 1 to 10), while during melting, the QC loses these facets and becomes increasingly rounded. Discontinuities in the calculation of interfacial velocity for facet 1 and 10 are due to the fact that the QC grows out of the tomographic FOV when it is largest. Scale bar measures 50 µm. (**c**) Average normal velocity of each facet. The analyses presented in (**a**,**b**) have been extended to the full QC volume in Fig. S1. (**d**) Area fraction of all ten aperiodic {10000} facets over the total surface area of the QC.

Isochrones of the solid-liquid interface in the ten-fold, {00001} plane (corresponding to the grey line in Fig. 2) are shown in Fig. 3 with 80 sec increments. The interfaces are colored according to their local, interfacial velocity, which was calculated using the nearest-neighbor approach given by Shahani *et al*.^40^ (see Methods). We follow the thermodynamic convention, wherein positive velocity indicates growth, and negative velocity indicates melting. In the initial stage of growth, facets are not readily distinguishable at the resolution of XRT. Nevertheless, the facets become more apparent as the QC grows: The fourth isochrone (1040 sec) from the center in Fig. 3(a) visibly displays the ten facets (numbered). After the QC is fully faceted, its growth is nearly isotropic, i.e., the growth velocity is almost uniform across all ten facets. Any differences in the facet velocities during growth are most likely due to thermosolutal convection. During melting (Fig. 3(b)), however, the interfacial velocity is faster at the bottom of the QC (near facet 10) than the top (near facet 5). Due to the anisotropic nature of melting, the QC surface loses its facets and becomes increasingly rounded as a function of time. Figure 3(c) depicts the average velocity of each QC facet in time. Consistent with Fig. 3(a), the velocities are almost the same for all ten facets during growth. However, during melting (i.e., after 1520 sec) there is a marked deviation in the facet velocities, which in turn depends on the facet orientations. The facets at the bottom melt faster than the facets at the top (cf. Figure 3(b)). We have also quantified the proportion of the area covered by the ten {10000} facets, over the total surface area, see Fig. 3(d). This was done by examining the ten peak intensities within ~14° windows in the stereographic projection of the interface normals, as a function of time (Methods). Consistent with Fig. 3(a,b), the area fraction of {10000} increases during growth and decreases during melting. Regardless of the peak window size, we note that this trend remains qualitatively the same.

### Analysis of liquid phase compositions

In addition to the morphology and dynamics of the QC, we measured the time-dependent composition of the liquid phase during solidification. This was done by analyzing the variation of the intensity in the X-ray projection images. In an absorption contrast X-ray imaging experiment, a higher intensity is typically associated with low atomic number elements in the microstructure due to less attenuation of the incident beam^36^. Recall that in our experiment, non-congruent growth (i.e., rejection of Al) gave rise to X-ray absorption contrast and induced an intensity difference. Put more quantitatively, using a monochromatic source, Husseini *et al*. proved that the intensity should vary linearly across the X-ray projection image for small, linear changes in atomic fraction^41^. Thus, the observed intensity can be directly mapped to composition, provided that the projection intensity has been calibrated against some features in the microstructure of known composition^42^. As demonstrated by Becker *et al*.^43^, this approach is viable for *both* monochromatic and polychromatic sources. For instance, Becker *et al*. calculated the composition of an Al-Ge alloy using a laboratory-based polychromatic X-ray source by calibrating the projection intensity against two liquid alloys of different compositions^43^. Here, we used the following two features in the microstructure to correlate projection intensity (a.u.) to composition (at%):

1. The solid QC phase. Note the proportion of Al atoms to the heavy atoms (Ni and Co) in the QC is 7:3 at any temperature according to the phase diagram (Fig. 1(b))^35^. The projection intensity of the QC along <00001> is denoted “2” in Fig. 4(a). The average projection intensity is measured when the QC is viewed “end-on,” i.e., there are no pockets of liquid in the path of the beam within region “2”.

**Figure 4:**
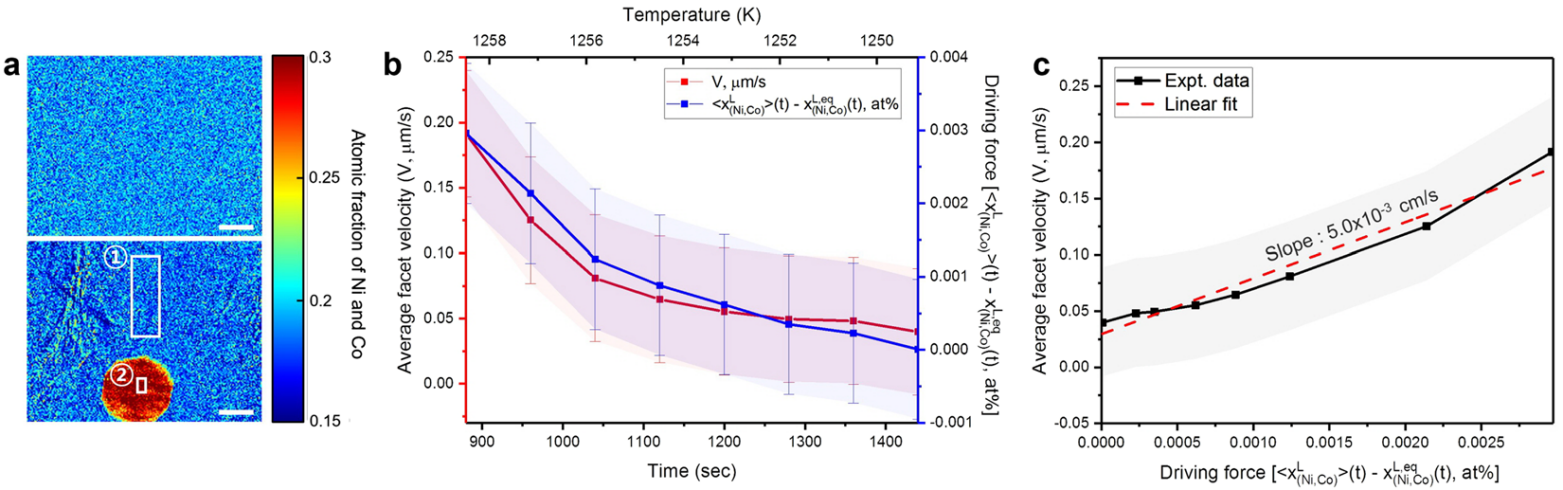
*Analysis of time-dependent driving force*. (**a**) X-ray projection images collected at 1272.5 K (40 sec, top image) and 1247.8 K (1520 sec, bottom image) during continuous cooling, respectively. The region contained in the first white box (“1”) was used to calibrate the average intensity from the liquid, $< {{\rm{x}}}_{{\rm{Co}},{\rm{Ni}}}^{{\rm{L}}} > ({\rm{t}})$, and the second white box (“2”) was used to calibrate the average intensity from the QC. The wrinkles in the bottom image are due to the thin oxide skin. Scale bars measure 100 µm. (**b**) Average facet velocity of the ten quasicrystalline facets of the decagonal QC (red) and kinetic driving force (blue), during the growth process. The driving force of supersaturation was calculated by subtracting the equilibrium liquid composition from the instantaneous liquid composition, see text and equation (2) for details. Errors in the measurement of average facet velocity are due to small errors in segmentation while those in the calculation of driving force are attributed to errors in the calibration of the phase compositions at equilibrium. (**c**) Average facet velocity *vs*. driving force. The slope gives the kinetic coefficient *β*_*s*_ associated with the growth process.
2. The liquid phase at equilibrium. At the instance that the QC stops growing, the supersaturation (i.e., a driving force for crystal growth) in the liquid phase must be equal to zero and hence the liquid phase is at equilibrium. In this case, the composition of the liquid phase can be read directly from the phase diagram (Fig. 1(b))^35^, since the alloy temperature is known. The projection intensity of the liquid phase at equilibrium is denoted “1” in Fig. 4(a). For comparison, we also show the highly supersaturated liquid phase prior to QC nucleation, directly above it.

In this manner, we calculated the average, time-dependent composition of the liquid phase, which we write as $< {{\rm{x}}}_{{\rm{Ni}},{\rm{Co}}}^{{\rm{L}}} > ({\rm{t}})$, during the growth process. Note that it is impossible to decouple the contributions of Ni and Co within $< {{\rm{x}}}_{{\rm{Ni}},{\rm{Co}}}^{{\rm{L}}} > ({\rm{t}})$ using only the above two conditions; to do so would require a third such condition. Thus, $< {{\rm{x}}}_{{\rm{Ni}},{\rm{Co}}}^{{\rm{L}}} > ({\rm{t}})$ represents only the total atomic fraction of the heavy elements Ni and Co in the liquid phase at a particular instance in time. Our calculation of $< {{\rm{x}}}_{{\rm{Ni}},{\rm{Co}}}^{{\rm{L}}} > ({\rm{t}})$ is still valuable as it permits us to measure the time-dependent supersaturation during QC growth: For instance, when $< {{\rm{x}}}_{{\rm{Ni}},{\rm{Co}}}^{{\rm{L}}} > ({\rm{t}})$ is much greater than the equilibrium liquidus composition given by ${\,x}_{{\rm{Ni}},{\rm{Co}}}^{{\rm{L}},{\rm{equil}}.}({\rm{t}})$, the liquid phase is highly supersaturated in the heavy elements Ni and Co. This situation occurs immediately following QC nucleation.

The precise relationship between supersaturation and QC velocity will be dealt with below.

## Discussion

### Growth in aperiodic directions

In general, the morphology of a growing crystal results from an interplay of interfacial kinetics and bulk transport. The fact that the interfacial velocity is approximately the same for the ten <10000> aperiodic directions at each time interval during growth (see Fig. 3(a)) — irrespective of the physical location of these facets in the laboratory frame — suggests that facet motion is largely governed by *interfacial mobility* rather than *bulk transport*. To lend quantitative support for this claim, we applied transition-state theory as follows: During interface-limited growth, the growth rate is limited by clusters incorporating into the QC at the solid-liquid interface, wherein the clusters must overcome an activation energy barrier^44–46^. In this case, the growth rate V is proportional to the difference in a forward flux J_L→QC_ and a reverse flux J_QC→L_ at the interface as

1 $${\rm{V}}\propto {{\rm{J}}}_{{\rm{L}}\to {\rm{Q}}{\rm{C}}}-{{\rm{J}}}_{{\rm{Q}}{\rm{C}}\to {\rm{L}}}$$

For crystal growth to occur, J_L→QC_ > J_QC→L_, whereas at equilibrium J_L→QC_ = J_QC→L_ and hence V = 0. If the growth occurs under weak supersaturation, one can show that equation (1) becomes^45,46^

2 $${\rm{V}}({\rm{t}})={\beta }_{s}\,{[ < {{\rm{x}}}_{{\rm{Co}},{\rm{Ni}}}^{{\rm{L}}} > ({\rm{t}})-{{\rm{x}}}_{{\rm{Co}},{\rm{Ni}}}^{{\rm{L}},{\rm{equil}}}({\rm{t}})]}^{n}$$

where $< {{\rm{x}}}_{{\rm{Co}},{\rm{Ni}}}^{{\rm{L}}} > ({\rm{t}})$ and ${{\rm{x}}}_{{\rm{Co}},{\rm{Ni}}}^{{\rm{L}},{\rm{equil}}}({\rm{t}})$ are the *instantaneous* and *equilibrium* compositions of the liquid phase, respectively; the square-bracketed term represents the supersaturation (i.e., driving force) that is required for crystal growth to occur; *n* is an integer exponent that indicates the mechanism of interfacial attachment; and *β*_*s*_is a constant-of-proportionality known as the kinetic coefficient in a supersaturated (*s*) matrix. For example, interface kinetics of first order (*n* = 1), otherwise known as the “normal” growth mechanism, signifies growth of atomically rough interfaces decorated with terraces, ledges, and kinks, while interface kinetics of second order (*n* = 2) indicates spiral growth along a screw dislocation. Descriptions of other growth laws (*n* = 3 and so on) can be found in Tiller^47^.

In practice, we measured $< {{\rm{x}}}_{{\rm{Co}},{\rm{Ni}}}^{{\rm{L}}} > ({\rm{t}})$ directly from the X-ray projection images as a function of time and $< {{\rm{x}}}_{{\rm{Co}},{\rm{Ni}}}^{{\rm{L}},{\rm{equil}}}({\rm{t}}) >$ from the as-calculated phase diagram^35^. Hence, our *in situ* imaging experiment provides a unique window into the time-dependent driving force associated with QC growth. To simplify the analysis, we assumed that the variation of the kinetic coefficient *β*_*s*_is negligible within the temperature range of 1259.8 K to 1247.8 K at which growth occurs, and so *β*_*s*_ is considered to be a constant value. If the model presented in equations (1–2) is satisfied, the QC growth process is dominated by the kinetics of interface attachment in the regime of weak supersaturation. As shown in Fig. 4(b), the trends in interfacial velocity and driving force are comparable to each other during the growth process. By fitting the time-dependent growth velocity *vs*. driving force data (Fig. 4(c)) to a function of the form given in equation (2), we calculated the kinetic coefficient *β*_*s*_ and the temporal exponent *n* as 5.0 × 10^–3^ cm s^−1^ and 1.0021, respectively. An R^2^ value of 0.963 was obtained, indicating a good fit of the linear model to the experimental data. Thus, the growth of the QC in the <10000> aperiodic directions follows first-order kinetics. In a similar sense, refs^6,7^ suggest that the Al-Pd-Mn icosahedral QCs grow from an undercooled liquid phase *via* an interface-limited, first-order growth mechanism. In addition, we can convert our value of *β*_*s*_ = 5.0 × 10^−3^ cm s^−1^ to the more widespread *β*_*m*_ = 2.5 × 10^−6^ cm s^−1^ K^−1^, which represents the kinetic coefficient in an undercooled melt (*m*). In converting from one form of the coefficient to the other, we have made use of the slope of the liquidus curve in Fig. 1(b,c).

There are explicit geometric models of crystal growth when growth is predominantly governed by local interface kinetics^48–50^. For instance, due to the Frank-Chernov construction^51,52^, a crystal asymptotically approaches its kinetic Wulff shape that is bounded by the slowest-moving facet planes during growth^53^. That is, the growth shape is determined solely by the locally-controlled orientation-dependent normal growth velocity. From visual inspection, the initial observable shape of the QC (i.e., the innermost isochrone in Fig. 3(a)) also evolves toward its kinetic Wulff shape. Figure 3(d) provides quantitative evidence since the proportion of the slow-moving {10000} facets increases until the structure is fully bounded by ten distinguishable facets, thereby indicating its morphological evolution towards the kinetic Wulff shape.

Our results indicate that there must be attachment sites available on the kinetic Wulff shape — that is bounded by aperiodic {10000} interfaces — for the normal growth mechanism to be sustained. Otherwise, the kinetic coefficient *β*_*s*_ and hence the growth rate V would tend to zero^54^. Consistent with this argument, we determined that the Jackson *α*-factor^46^ for the decagonal QC phase is at most 0.14 from differential scanning calorimetry (DSC, see Fig. S2). The relatively low value (i.e., *α* < 2) indicates that the QC interfaces are atomically rough, similar to other solid metals grown from the liquid phase, e.g., Al and Ni^46^. Further support comes from a number of scanning tunneling microscopy (STM) studies by Thiel and coworkers^55–57^ that depict the complex surface structures of these aperiodic interfaces: For instance, the atomically-clean, five-fold facets of an icosahedral quasicrystal exhibit a terrace-step morphology, wherein the terraces are separated by steps of unequal heights. Moreover, icosahedral quasicrystals present multiple types of adsorption sites (e.g., “dark stars” and “white flowers”)^56^ due to the diverse range of atomic configurations. Thus, the aperiodic facets of a quasicrystal are not truly flat in the classical sense but possess a remarkable degree of heterogeneity.

The magnitude of the kinetic coefficient also merits further discussion. According to Chernov^58^, Markov^54^, and Land *et al*.^59^, the kinetic coefficient has a “steric” character: It is inversely proportional to the size of the attaching species because a larger species moves more slowly than a smaller one. Moreover, it takes more time for the larger species to rotate to the correct orientation^59^. In this way, the QC clusters should overcome a configurational entropy-type barrier^58^ in order to incorporate into the solid QC phase. Using this logic, one might expect that the kinetic coefficient of an aperiodic crystal — that is built from the attachments of large clusters — to be smaller than that of other simple substances. In this way, the kinetic coefficient could serve as a measure of structural complexity. Indeed, as shown in Table. S1, the kinetic coefficients *β*_*m*_of QCs — including Al-Cu-Fe^60^, Al-Pd-Mn^6,60^, and Al-Ni-Co (this work) — are all significantly smaller than those of periodic, elemental metallic crystals by approximately six to nine orders of magnitude, and those of periodic, intermetallic crystals by two to eight orders of magnitude. The kinetic coefficients listed for aperiodic crystals were determined from X-ray radiographs^6^ and a combination of the Avrami approach and differential thermal analysis^60^. Thus, it is entirely plausible that a higher configurational entropy-type barrier contributes to a more sluggish growth rate.

### Growth in periodic direction

Our results indicate a stark contrast in the growth of the QC along the periodic and aperiodic directions. The growth rate along the periodic <00001> direction is about two orders of magnitude greater than the growth rate in the aperiodic <10000> directions, and this anisotropy in the interfacial velocities results in an elongated shape of a decagonal prism, see Fig. 2. To explain these trends, higher order kinetics deserves consideration since the degree of supersaturation is nearly uniform around the QC. According to a study^61^ of an Al-Cu-Co single decagonal quasicrystal by X-ray topography, contrast associated with a screw dislocation appears in the {00001} plane. This suggests that growth along <00001> occurs *via* second order kinetics wherein the interfacial velocity is related to the square of the driving force (i.e., equation (2) with *n* = 2). The spiral ledges provide enough sites for crystal growth and thus there is no need of ledge nucleation. Consequently, the growth rate of crystal along the periodic direction is likely to be faster than in the aperiodic directions, even with a small amount of supersaturation^44–46^. Future improvements in higher resolution dynamic imaging would provide more conclusive support of this mechanism.

### Melting

During melting, the QC loses its faceted solid-liquid interfaces, achieving a more rounded morphology (cf. Fig. 3(b)). Mechanistically, this may occur if the weakly bound, corner clusters leave the solid as soon as melting is initiated, further exposing new corners and perpetuating the melting process. The resulting, smooth curvature of the solid-liquid interface upon melting suggests that the removal of clusters occurs in a continuous way instead of in abrupt jumps, as implied by transition-state theory (above). That is, melting is not an activated process, in contrast to growth. As a result, the melting process does not follow the geometric models mentioned above because melting is dominated by non-local, transport processes, as will be further explained below. If QC melting occurred via a local mechanism, the corners should evolve into facets, as has been observed by Wettlaufer and coworkers for a twelve-sided snowflake^62^. However, this behavior is not seen here (Fig. 3(b)).

Importantly, the rate of melting is not the same for each of the ten corners, otherwise the isochrones of the solid-liquid interface would be perfectly circular. Rather, the melting velocities depend strongly on the *physical* orientation, and not the *crystallographic* orientation. As a result, the melting process is asymmetric. For instance, after 1520 sec, facets 1, 9, and 10 show greater velocities than other facets, see Fig. 3(b,c). Due to the influence of gravity, which points downward in Fig. 3, the heavy-atom Ni and Co-rich clusters at the bottom of the QC are removed more easily than at the top of the QC. The detached clusters tend to sink in the Al-rich liquid. Hence, the composition of heavy elements, i.e., Ni and Co, near the bottom interface of the QC decreases, while the composition of heavy elements near the top interface accumulates during the melting process. In other words, there exists a density difference between the top and bottom facets of the QC that gives rise to buoyancy-driven convection. Consequently, the bottom facet is brought into contact with an Al-rich liquid that, in turn, causes it to melt faster. Thus, transport kinetics are responsible for the unusual “egg-like” morphology of the QC at late times.

The increase in the interfacial velocity of the bottom-most QC facets as melting progresses suggests an unstable heavy elements distribution adjacent to these facets in the liquid phase, see Fig. 3(b,c). A similar behavior was recently observed *via in situ* X-ray radiography for Sn-Bi dendrites^63^ that were solidified parallel to gravity, in which the heavy elements formed large plumes ahead of the growth front. These plumes disturbed the stability of the growing dendrites, such that their growth velocity was highly inconsistent over time. Taken altogether, interfacial kinetics brings about crystalline order while gravity-induced convection leads to microstructural heterogeneity, in both periodic crystals and QCs alike.

## Conclusion

To the best of our knowledge, we performed the first-ever 4D X-ray tomography experiment on the evolution of the decagonal Al-15at%Ni-15at%Co QC phase upon growth and melting in an alloy of composition Al-9.55at%Ni-9.55at%Co. By extracting morphological, dynamic, and compositional information directly from our space- and time-resolved data, we were able to provide a fresh lens on these poorly understood phase transitions. On the basis of our results, we determined that the decagonal QC grows *via* a normal growth mechanism, similar to periodic crystals with atomically “rough” facets. Even so, the growth rate is remarkably sluggish compared to those of periodic crystals due to the rearrangement of clusters at the solid-liquid interface. To quantify the growth kinetics, we calculated an interface kinetic coefficient of 2.5 × 10^−6^ cm s^−1^ K^−1^, that is independent of the magnitude of the driving force. Hence, this coefficient can be used as input for mesoscale models (e.g., phase field simulations). On the other hand, melting is not an activated process and is governed purely by gravity-driven convection. We expect these experimental techniques and computational analyses will improve our understanding of kinetic phenomena that occur on the solid QC-liquid interface and, more generally, help researchers decipher the complex, multicomponent microstructures that arise during materials processing.

## Methods

### Experimental methodology

High purity (99.999% Al, 99.9% Ni and 99.9% Co) alloy samples of nominal composition Al-9.55at%Ni-9.55at%Co were prepared with the vacuum arc remelting (VAR) technique at the Materials Preparation Center (MPC) at Ames National Laboratory in Ames, IA, United States. The cast alloy buttons were cut into a rod of 1 mm diameter by 5 mm height for the synchrotron experiment.

XRT was performed at sector 2-BM at the Advanced Photon Source (APS) at Argonne National Laboratory in Lemont, Illinois, United States. The sample was first held above the liquidus temperature (1293.2 K) for approximately five minutes, to ensure complete melting. The molten alloy sample was protected by a thin oxide skin surrounding the sample, naturally grown by thermal oxidation. Once homogenized, the sample was then cooled continuously for one hour at a rate of 1 K min^−1^. One QC was observed between 1259.8 K and 1233.8 K during the scan, see text for details. Note the furnace temperature was calibrated against the established melting temperatures of other Al-based alloys, e.g., Al-Si and Al-Ge, prior to this experiment. Concurrent with the cooling process, X-ray projections were acquired at a rate of 50 Hz using a polychromatic “pink” beam centered at 27 keV, and a PCO Edge 5.5 CMOS camera that was optically coupled to a 20 µm LuAg:Ce scintillator. The FOV measured 2560 by 800 pixels, resulting in a pixel size of 0.65^2^ µm^2^. For each 180° rotation of the sample, 1000 frames were collected with an exposure time of 14 ms. Thus, the temporal resolution between consecutive 3D reconstructions was 20 sec.

Following XRT at Argonne, further experiments were conducted at the University of Michigan: For the transmission electron microscopy (TEM) analysis, an alloy sample of composition Al-10.5at%Ni-12.5at%Co was first prepared *via* VAR, as above. It was then ground and diluted in ethanol. TEM images and electron diffraction patterns were recorded at the Michigan Center for Materials Characterization ((MC)^2^) using the JEOL 2010F AEM with a double-tilt holder and 200 kV of accelerating voltage. For the DSC analysis, we investigated an Al-8at%Ni-8at%Co rod of 1 mm diameter by 5 mm height and 13.8 mg weight. The DSC analysis was performed in a nitrogen atmosphere using TA Instrument DSC SDT Q600 with a 10 K min^−1^ heating rate. The temperature range was from 290.9 K to 1295.4 K. See Fig. S2.

### Data processing

**Figure 5:**
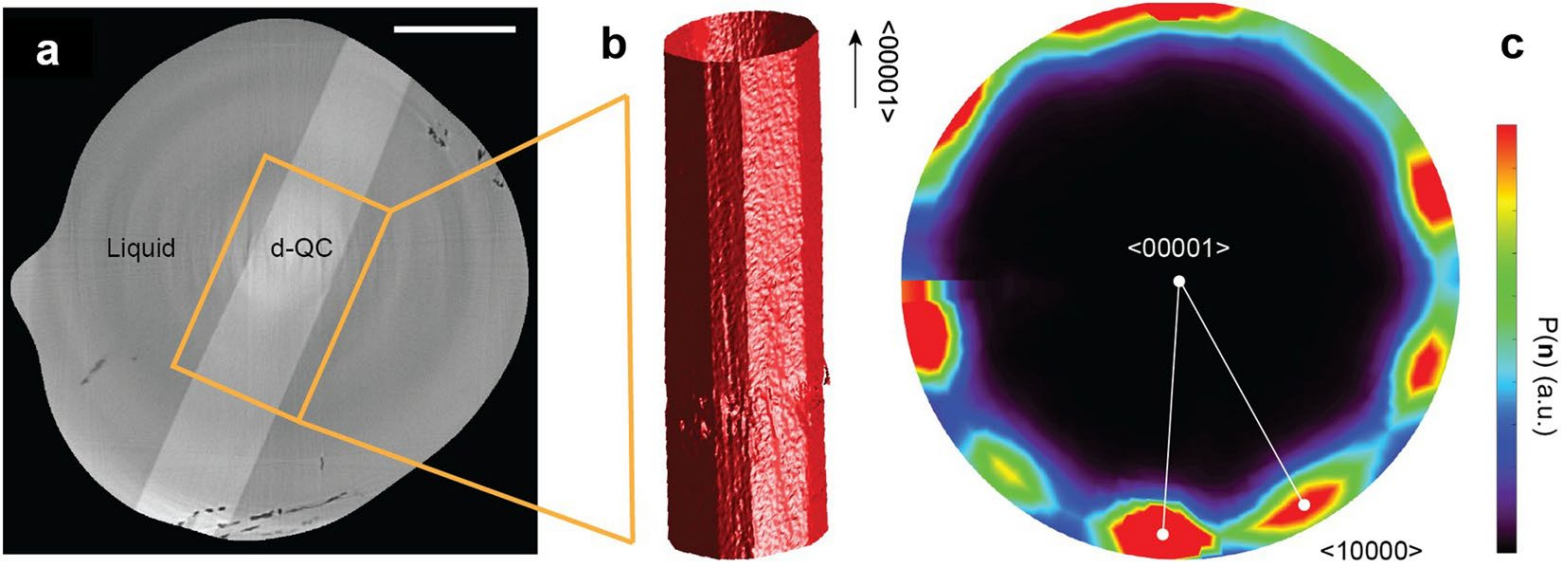
*Data processing pipeline*. (**a**) 2D slice of a 3D reconstruction, showing the clear contrast between the decagonal QC (in light grey) and the liquid phase (in dark grey). The scale bar measures 250 μm. (**b**) 3D QC corresponding to the highlighted section in (**a**). Faceted interfaces are evident. (**c**) Stereographic projection of the local interfacial normals, taken along the <00001> zone axis. Consistent with a decagonal morphology, the projection shows ten peaks separated by approx. 36°. The colorbar indicates the probability $P$ of finding a particular orientation $\hat{n}$ along the solid-liquid interfaces.

The tomographic data was reconstructed with TomoPy^64^, a Python-based open source framework for the analysis of tomographic data. Within TomoPy, we first normalized the X-ray projections by the dark-field and white-field images. Normalization alone was not sufficient to correct for the “ring”-shaped artifacts, which are typically caused by a combination of dead pixels in the CCD as well as beam instabilities. Such artifacts were corrected here *via* a combined wavelet-Fourier filter^65^. After normalization and artifact removal, the data were reconstructed *via* the Gridrec algorithm^66^, which is a direct Fourier-based method. More details on these algorithms can be found in ref.^64^ and the references therein. A representative slice of the reconstructed volume (along the axis of rotation) is given in Fig. 5(a). The strong absorption contrast allows one to easily distinguish between the solid decagonal QC (light grey) and the liquid phase (dark grey).

Further data processing was conducted using the Image Processing toolbox in MATLAB R2016b. Specifically, we subtracted the reconstruction of the fully-liquefied sample from all other reconstructions, in order to enhance the contrast between the solid and liquid phases. In addition, we applied basic morphological operations (e.g. dilation) on each 2D reconstruction slice, so as to suppress any remaining speckle noise. The processed 2D images were then segmented with an appropriate threshold, and combined to reveal the 3D microstructures. For the subsequent analysis, the solid-liquid interfaces were meshed, or represented by a sequence of triangles and vertices^67^. To remove any staircasing artifacts, we smoothed the triangular mesh *via* mean curvature flow^68^. An example of a smoothed mesh is shown in Fig. 5(b). The following calculations make use of the mesh face and vertex positions. We denote the three vertices of triangle face *i* as ${v}_{1}^{i}$, ${v}_{2}^{i}$, and ${v}_{3}^{i}$.

### Microstructural analysis

We quantified QC growth and melting by computing the local interfacial orientation and local velocity of each triangle in the mesh. Beyond these two metrics, we derived the local composition of the “heavy” elements directly from the normalized X-ray projection images, as described below. The local interfacial orientation $\hat{n}$ was used to assign each of the mesh triangles to one of the ten facets of the QC. Given that the edges of triangle face *i* are defined as ${\vec{e}}_{12}^{i}={v}_{2}^{i}-{v}_{1}^{i}$, ${\vec{e}}_{23}^{i}={v}_{2}^{i}-{v}_{3}^{i}$, and ${\vec{e}}_{31}^{i}={v}_{3}^{i}-{v}_{1}^{i}$, and that the vertex order is consistent for all faces, the unit normal vector $\hat{n}\,\,$can be found as

3 $$\hat{n}=({\vec{e}}_{12}^{i}\times {\vec{e}}_{23}^{i})/|{\vec{e}}_{12}^{i}\times {\vec{e}}_{23}^{i}|$$

Following thermodynamic convention, all normal vectors point from the QC to the liquid. Geometrically, the denominator in equation (3) represents the area of triangle face *i*, ${A}^{i}=|{\vec{e}}_{12}^{i}\,\times \,{\vec{e}}_{23}^{i}|$. After computing all normal vectors, we next plotted the interface normal distribution (IND)^69^, which indicates the degree of directionality in the microstructure. In practice, we depicted the IND as a stereographic projection of the interface normal vectors $\hat{n}$ along the <00001> axis, see Fig. 5(c). While the normal vectors were measured in the laboratory frame, it was relatively easy to identify the periodic “long axis” from direct inspection (Fig. 5(b)). The IND reveals the ten-fold symmetry of the decagonal QC in the same way as the electron diffraction (ED) pattern in Fig. 1(b). However, the two representations are different in the sense that the IND conveys the *extrinsic* directionality of the faceted solid-liquid interfaces while ED conveys the *intrinsic* lattice symmetry. The IND at each time-step was then segmented with an appropriate threshold to give those mesh triangles that correspond to the {10000} aperiodic facets. In this way, we clustered a set of mesh triangles into ten facets. A similar procedure was employed by Shahani *et al*.^70^ to determine the mesh triangles oriented along {111} in polycrystalline Si.

In addition to the local interfacial orientation, we calculated the local interfacial velocities according to a nearest neighbor (NN) algorithm specified by Shahani *et al*.^71^. In short, we found the NN vertex in the mesh corresponding to time-step *t* + Δ*t*, for each face centroid at time $t$. The distance between these two points divided by the time interval Δ*t* gives the face velocity ${V}^{i}$. In Fig. 3, however, we report not the local *face* velocity but the collective *facet* velocity, which can be found as

4 $${V}^{facet}=\sum _{i\,{\epsilon }\,facet}{A}^{i}{V}^{i}/\sum _{i\,{\epsilon }\,facet}{A}^{i}$$

Thus the facet velocity is a weighted average of the local triangle face velocities. The summations in equation (4) are carried out only for those triangle faces *i* that belong to the facet. The facet velocities have been determined for the entire 3D QC, see Fig. S1.

### Compositional analysis

The intensity ***I*** of a transmitted X-ray beam depends on many factors, including sample thickness, chemical composition, and beam hardening. For example, ${\boldsymbol{I}}\propto {{\boldsymbol{e}}}^{-{\boldsymbol{d}}}$ according to the Beer-Lambert law, where ***d*** is sample thickness. Thus, to ensure that differences in intensity reflect only the differences in chemical composition — such that the composition mapping strategy described in the Results can be applied — the following precautions were taken: First, we selected the same thicknesses of liquid and QC (approx. 1110 µm), in regions 1 and 2 respectively (Fig. 4(a)). In addition, we subtracted a projection image of the fully liquefied sample at 1273.2 K from every projection that followed. This was done to mitigate the effect of beam hardening, which arises when a polychromatic X-ray beam becomes “harder” due to the ease of absorption of soft X-rays.

After these processing steps, we measured the average transmitted X-ray intensities from the QC and liquid at equilibrium (1247.8 K). Correspondingly, the composition of the QC and liquid at equilibrium are read from the phase diagram^35^. Thus, we can simply solve the simultaneous equations that give the contributions of Al and heavy elements (Ni and Co) to X-ray intensity. In this manner, the composition of the liquid phase, in terms of Al and the heavy elements, is attained for all time-steps. Additionally, to determine the total alloy composition $< {{\bf{x}}}_{{\bf{C}}{\bf{o}},{\bf{N}}{\bf{i}}}^{{\bf{t}}{\bf{o}}{\bf{t}}{\bf{a}}{\bf{l}}} > ({\bf{t}})$ in the FOV, as plotted in Fig. 1(c), we need to know the QC volume fraction, ***f*** ^***QC***^, which is simply found from the segmented 3D reconstructions as the sum of all voxels belonging to the QC phase divided by the sum of all voxels contained in the rod sample within the FOV. Then, $< {{\bf{x}}}_{{\bf{C}}{\bf{o}},{\bf{N}}{\bf{i}}}^{{\bf{t}}{\bf{o}}{\bf{t}}{\bf{a}}{\bf{l}}} > ({\bf{t}})$ can be found from the lever rule as

5 $$ < {{\rm{x}}}_{{\rm{Co}},{\rm{Ni}}}^{{\rm{total}}} > ({\rm{t}})={f}^{QC}{{\rm{x}}}_{{\rm{Co}},{\rm{Ni}}}^{{\rm{QC}}}+(1-{f}^{QC}) < {{\rm{x}}}_{{\rm{Co}},{\rm{Ni}}}^{{\rm{L}}} > ({\rm{t}})$$

where the term (1−***f*** ^***QC***^) represents the volume fraction of the liquid phase.

### Data availability

XRT projection data are stored on the Materials Data Facility repository^72^ and are publically available at http://dx.doi.org/10.18126/M2K910.

## Electronic supplementary material

Supporting information
