# Supplementary material for: Probing the growth and melting pathways of a decagonal quasicrystal in real-time
Source: Sci Rep. 2017 Dec 12;7:17407. doi: 10.1038/s41598-017-17821-0 (PMC5727210; doi:10.1038/s41598-017-17821-0)
Supplement: Supplementary file 1 — Supporting information [file 41598_2017_17821_MOESM1_ESM.pdf]

Probing the growth and melting pathways of a decagonal quasicrystal in real-time

Supporting information

Insung Han[1,*], **Xianghui Xiao**[2], **and Ashwin J. Shahani**[1,*]

[1]Department of Materials Science and Engineering, University of Michigan, Ann Arbor, Michigan, 48109, United States
[2]X-ray Science Division, Advanced Photon Source, Argonne National Laboratory, Lemont, Illinois, 60439, United States
[*]corresponding authors: I.H. (email: insungh@umich.edu) and A.J.S. (email: shahani@umich.edu)

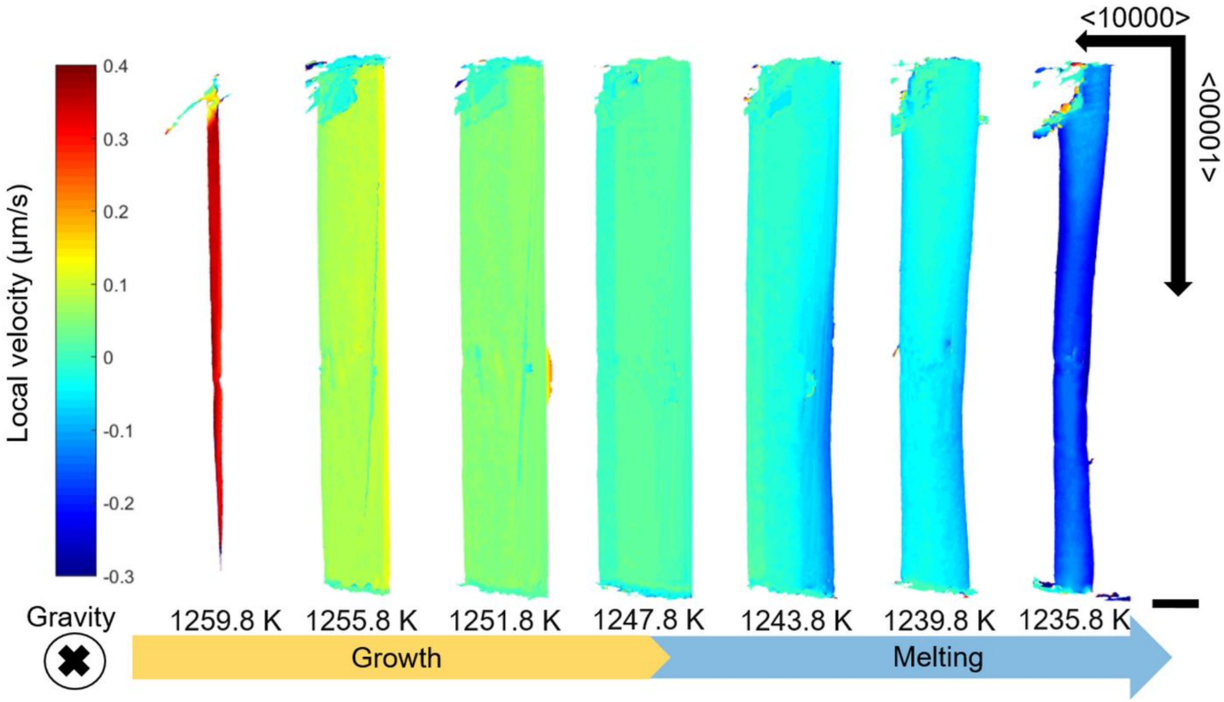

Fig S1. *3D reconstruction of full volume of QC*, wherein the interfaces have been colored according to local velocity. The temperature decreases from left to right as a function of reaction time. Temperatures and times are 1259.8 K (800 sec), 1255.8 K (1040 sec), 1251.8 K (1280 sec), 1247.8 K (1520 sec), 1243.8 K (1760 sec), 1239.8 K (2000 sec), and 1235.8 K (2240 sec), respectively. Scale bar measures 100 μm. The local velocity is roughly constant during growth, suggesting that the growth process is dominated by interfacial attachments and is scarcely affected by external flows. On the other hand, the interfacial velocity varies not with *crystallographic* orientation but with *physical* orientation during melting, suggesting that it is instead driven by thermosolutal convection. See text for details.

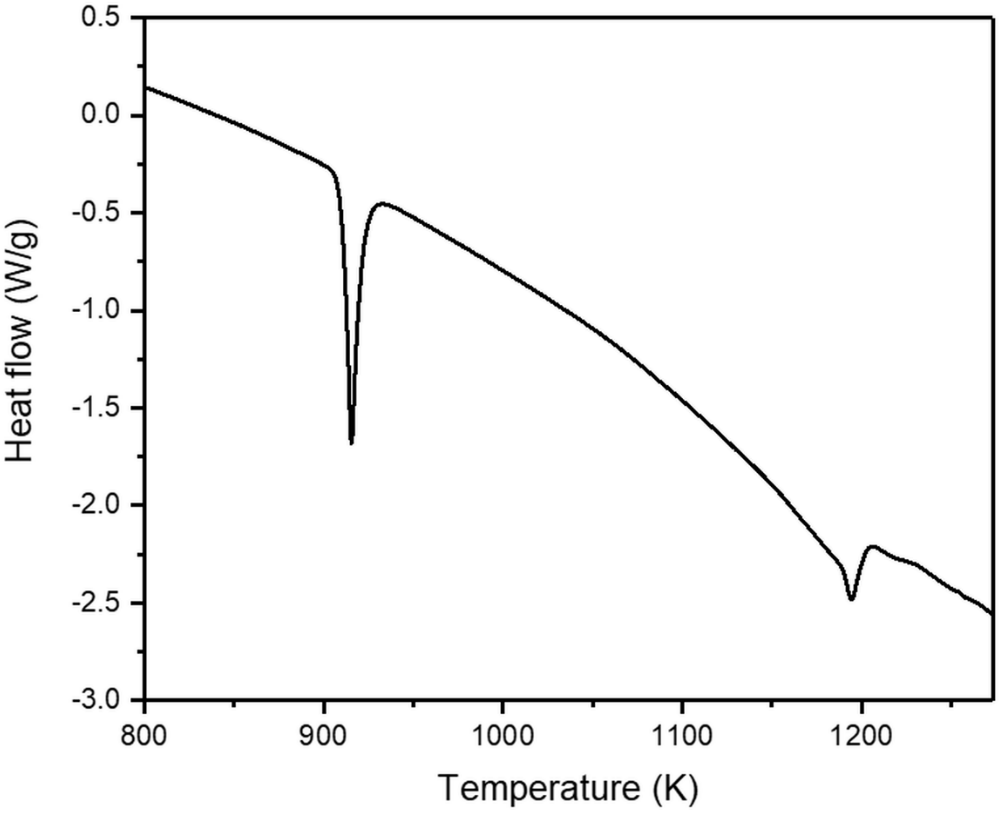

Fig. S2. *Differential scanning calorimetry* (DSC) data collected on the Al-8at%Ni-8at%Co phase, upon heating. The two sharp peaks correspond to the phase transitions, $Al + Y \rightarrow L + Y$ (915.4 K) and $L + D \rightarrow L$ (1194.2 K), respectively. Y denotes the periodic approximant phase, see **Fig. 1(b)**. The latent heat of fusion ΔH_f was calculated as 1.43 x 10^3 J mol^{-1} from the second sharp peak at 1194.2 K, from which the entropy of fusion ΔS_f and an upper estimate of the Jackson α-factor were determined as 1.20 J $mol^{-1}K^{-1}$ and 0.14, respectively. Here, $\alpha \leq \Delta S_f/R$ where R is the ideal gas constant.

**Material**	**Kinetic coefficient, β_m (cm s^{-1} K^{-1})**	**Reference**
Au	36.3 {100}<br>20.7 {110}<br>10.1 {111}	Hoyt *et al.*[1]
Ni	52 {100}<br>40 {110}	Hoyt *et al.*[2]
Fe	30.5 {100}<br>25.7 {110}	Watanabe *et al.*[3]
Cu	46 {100}<br>27 {110}<br>19 {111}	Hoyt *et al.*[4]
FeSi	1.4	Herlach[5]
CoSi	2.1	Herlach[5]
$Y_3Al_5O_{12}$ (YAG)	3.5×10^{-3}	Nagashio & Kuribayashi[6]
Al_2O_3	$\sim3.5\times10^{-2}$	Nagashio & Kuribayashi[6]
Al-Ni-Co d-phase	2.5×10^{-6} {10000}	This work
Al-Pd-Mn i-phase	9.0×10^{-5}<br>9.4×10^{-7}	Thi *et al.*[7]<br>Dong *et al.*[8]
Al-Cu-Fe i-phase	3.0×10^{-8}	Dong *et al.*[8]

Table S1. *Kinetic coefficients of undercooling in a liquid phase*, β_m, of various crystals, as determined from both experiments and simulations. The values of β_m of aperiodic crystals are all significantly smaller than those of periodic, elemental metallic crystals by approximately six to nine orders of magnitude, and those of periodic, intermetallic crystals by two to eight orders of magnitude, indicating a slower growth rate. The kinetic coefficient derived here for the decagonal (d) phase is consistent with other studies on the icosahedral (i) QC phase.

References

1 Hoyt, J. & Asta, M. Atomistic computation of liquid diffusivity, solid-liquid interfacial free energy, and kinetic coefficient in Au and Ag. *Phys. Rev. B* **65**, 214106 (2002).

2 Hoyt, J., Asta, M. & Karma, A. Atomistic simulation methods for computing the kinetic coefficient in solid-liquid systems. *Int. Sci.* **10**, 181-189 (2002).

3 Watanabe, Y., Shibuta, Y. & Suzuki, T. A molecular dynamics study of thermodynamic and kinetic properties of solid–liquid interface for bcc iron. *ISIJ int.* **50**, 1158-1164 (2010).

4 Hoyt, J., Sadigh, B., Asta, M. & Foiles, S. Kinetic phase field parameters for the Cu–Ni system derived from atomistic computations. *Acta Mater.* **47**, 3181-3187 (1999).

5 Herlach, D. M. Direct measurements of crystal growth velocities in undercooled melts. *Mater. Sci. Eng. A* **179**, 147-152 (1994).

6 Nagashio, K. & Kuribayashi, K. Rapid solidification of Y 3 Al 5 O 12 garnet from hypercooled melt. *Acta Mater.* **49**, 1947-1955 (2001).

7 Thi, H. N. *et al.* In situ and real-time probing of quasicrystal solidification dynamics by synchrotron imaging. *Phys. Rev. E* **74**, 031605 (2006).

8 Dong, C., Dubois, J., De Boissieu, M., Boudard, M. & Janot, C. Growth of stable Al–Pd–Mn icosahedral phase. *J. Mater. Res.* **6**, 2637-2645 (1991).
